# Gastric Cancer Tumor Microenvironment Characterization Reveals Stromal-Related Gene Signatures Associated With Macrophage Infiltration

**DOI:** 10.3389/fgene.2020.00663

**Published:** 2020-06-30

**Authors:** Shenyu Wei, Jiahua Lu, Jianying Lou, Chengwei Shi, Shaowei Mo, Yaojian Shao, Junjie Ni, Wu Zhang, Xiangdong Cheng

**Affiliations:** ^1^Department of First Clinical Medical College, Zhejiang Chinese Medical University, Hangzhou, China; ^2^Division of Hepatobiliary and Pancreatic Surgery, Department of Surgery, First Affiliated Hospital, School of Medicine, Zhejiang University, Hangzhou, China; ^3^NHC Key Laboratory of Combined Multi-organ Transplantation, Hangzhou, China; ^4^Key Laboratory of Organ Transplantation, Hangzhou, China; ^5^Department of Hepato-Pancreato-Biliary Surgery, The Second Affiliated Hospital, Zhejiang University School of Medicine, Hangzhou, China; ^6^Shulan Hospital Affiliated to Zhejiang Shuren University Shulan International Medical College, Hangzhou, China; ^7^School of Medicine, Zhejiang University, Hangzhou, China; ^8^Department of Abdominal Surgery, Zhejiang Cancer Hospital, Hangzhou, China

**Keywords:** gastric cancer, tumor microenvironment, tumor-associated macrophage, stromal score, bioinformatic, biomarker

## Abstract

The tumor microenvironment (TME) has attracted attention owing to its essential role in tumor initiation, progression, and metastasis. With the emergence of immunotherapies for various cancers, and their high efficacy, an understanding of the TME in gastric cancer (GC) is critical. The aim of this study was to investigate the effect of various components within the GC TME, and to identify mechanisms that exhibit potential as therapeutic targets. The ESTIMATE algorithm was used to quantify immune and stromal components in GC samples, whose clinicopathological significance and relationship with predicted outcomes were explored. Low tumor mutational burden and high M2 macrophage infiltration, which are considered immune suppressive characteristics and may be responsible for unfavorable prognoses in GC, were observed in the high stromal group (HR = 1.585; 95% CI, 1.112–2.259; *P* = 0.009). Furthermore, weighted correlation network, differential expression, and univariate Cox analyses were used, along with machine learning methods (LASSO and SVM-RFE), to reveal genome-wide immune phenotypic correlations. Eight stromal-relevant genes cluster (*FSTL1, RAB31, FBN1, ANTXR1, LRRC32, CTSK, COL5A2*, and *ENG*) were identified as adverse prognostic factors in GC. Finally, using a combination of TIMER database and single-sample gene set enrichment analyses, we found that the identified genes potentially contribute to macrophage recruitment and polarization of tumor-associated macrophages. These findings provide a different perspective into the immune microenvironment and indicate potential prognostic and therapeutic targets for GC immunotherapies.

## Introduction

Gastric cancer (GC) is the fifth most frequently diagnosed cancer and the third leading cause of cancer-related deaths worldwide (Bray et al., 2018). Advanced patients who are no longer eligible for surgery are forced to resort to other therapies. In the past five years, immunotherapy has emerged as the standard of care for many advanced cancers (Lordick et al., 2017). In GC, positive responses to immunotherapy are limited to a small fraction of patients, and, owing to tumor heterogeneity, its efficacy remains to be elucidated (Shi et al., 2012; Salati et al., 2019). Therefore, an understanding of immunotherapy mechanisms is a priority for the management and extension of positive responses to broader target populations.

The tumor microenvironment (TME) is a repertoire of ostensibly normal cells, recruited by cancer cells, that contribute to cancer initiation, growth, and dissemination (Hanahan and Weinberg Robert, 2011). Components of TME include fibroblasts, immune cells, endothelial cells, along with their secreted extracellular matrix (ECM) (Kobayashi et al., 2019). With the understanding of the diversity and complexity of TME in GC deepening, mounting evidence suggests its crucial role in tumor initiation, progression, immune evasion, and its effect on tumor response to immunotherapies (Lee et al., 2014). Prevalent infection of *Helicobacter pylori* in GC patients underlies a chronic inflammatory environment, which is considered a major risk for GC development (Polk and Peek, 2010). In addition, patients with intestinal metaplasia, gastric atrophy, and cancers demonstrated an increased incidence of genetic alterations strongly correlated with immune response (Lott and Carvajal-Carmona, 2018). Therefore, through the systematic analysis of the heterogeneity and complexity of GC TME, typical tumor characterization could be identified, and our ability for guiding and predicting immunotherapies could also be improved (Zeng et al., 2019). Previously, tumor immune infiltration was mainly studied using flow cytometry and immunohistochemistry (IHC), which require large amounts of tissues and high sample quality (Mellors, 1968; Melato, 1997). Nowadays, emerging computational methods are supporting these analyses and rapidly revealing a broader intra-tumoral immune landscape. Such methods are based on gene expression profiles and immunological features, which include Estimation of STromal and Immune cells in MAlignant Tumor tissues using Expression data (ESTIMATE) and Cell-type Identification By Estimating Relative Subsets Of RNA Transcripts (CIOBSORT) algorithms (Yoshihara et al., 2013; Newman et al., 2015).

Consequently, on this basis, we performed a multi-dimensional and multi-perspective analysis to reveal the potential relationship between immune infiltration and the genome in GC using various advanced bioinformatic algorithms. In this study, we used weighted gene co-expression network analysis (WGCNA) to build gene networks, through which the connection between corresponding genes are identified and weighted based on their expression. After transforming the expression profiles into weighted networks, the genes are clustered into modules with distinct clinical characteristics, in which the genes are highly co-expressed. Compared with direct screening of differentially expressed genes (DEGs), gene sets identified with current methods are more biologically connected and significant (Wang et al., 2019). Furthermore, machine leaning methods including support vector machine-recursive feature elimination (SVM-RFE) and least absolute shrinkage and selection operator (LASSO) algorithms, were adopted for the identification genes correlating with prognosis. The interactive employment of the two methods ensures the hub genes with best prognostic value and multiple characteristics. Additionally, the potential relationship between immune features and hub genes were subjected to single-sample gene set enrichment analysis (ssGSEA) for second validation, which greatly enhanced the reliability of our study.

Overall, we aimed to identify hub genes associated with GC TME by combining WGCNA with immune and stromal scores in The Cancer Genome Atlas (TCGA) database. Our results established an optimized combination of various advanced algorithms to identify TME-related genes and revealed potential mechanisms by which the TME-related genes promoted GC development. The workflow is summarized in Supplementary Figure S1.

## Materials and Methods

### Data Acquisition and Pre-processing

Gene expression data for 375 GC samples and 32 normal samples were downloaded as level 3 RNA-seq FPKM datasets from TCGA^1^. Ensembl IDs were converted into gene symbol matrices using online datasets^2^. Expression values for the same gene names were averaged. The mRNA expression matrix was extracted from standardized data. GC sample somatic mutation data were downloaded using the TCGAbiolinks R package and portal mutect2 (Colaprico et al., 2016). The maftools R package was used for mutational profile analysis and visualization. The corresponding clinical information from patients with GC, including data for age, gender, ethnicity, microsatellite instability, pathologic stage, histologic grade, survival status, and survival time were retrospectively collected from the TCGA database (Supplementary Table S1).

### Tumor Mutational Burden Calculation

TMB, the number of somatic missense mutations per megabase of a patient’s genome, is a potential biomarker for prediction of response to immunotherapy (Rizvi et al., 2015; Goodman et al., 2017). In this study, 38 Mb was used as the estimated whole exome size (Chalmers et al., 2017). We determined TMB using the equation: $\text{TMB}=\frac{S_{i}\times 1,000,000}{I}$ (*I* represents the number of exonic bases with a coverage depth ≥100×, and *S*_*i*_ represents the absolute number of somatic mutations).

### WGCNA

The genes with upper 25% median absolute deviation were selected to guarantee data heterogeneity and accuracy of WGCNA analysis. We calculated the Pearson correlation coefficient between each paired gene using their absolute transcript expression values, defined as: *L*_*ij*_ = |*cor*(*x*_*i*_,*x*_*j*_)| (*L* represents the Pearson correlation coefficient between gene *i* and *j*). We presented this value in a co-expression similarity matrix. We exponentiated the correlation coefficient to reflect the continuous nature of the genes with potential co-expression. To maintain scale independence and mean connectivity balanced in a topology network, we calculated thresholding powers from 1 to 20. An appropriate power value was selected to emphasize strong correlations and to penalize the weak ones to achieve a scale-free topologic network. Next, the weighted adjacency matrix was constructed in power operation: $a_{s\,t}=l_{s\,t}^{\beta }$ (where *a* is the adjacency between genes *s* and *t*). The topological overlap matrix (TOM) was converted from the adjacency matrix to reflect the sum of direct and indirect correlations between genes in the network. Then, we performed hierarchical clustering based on the TOM-based dissimilarity value (1-TOM) and obtained module dendrograms. The minimum genome size of the module was set as 40.

Gene significance (GS) represents the correlation between gene expression and clinical characteristics. Module eigengenes, the first principal components, were calculated to identify the gene expression signature landscape of each module. Module eigengenes were correlated with clinical traits in a heatmap. To merge similar modules and augment the capacity of the modules, the cutoff value was set as 0.25. Clinical characteristics were analyzed with the gene modules. After identifying modules most related with interesting phenotypes, GS was correlated with module membership (MM), defined as the similarity between a gene expression profile and the module in which it belongs.

### Functional Enrichment Analysis and Screening Differentially Expressed Genes

For genes in the module being examined, we used the clusterProfile R package for gene ontology functional annotations with a false discovery rate (FDR) threshold less than 0.05. The limma R package was used to identify genes aberrantly expressed in GC, with the selection criteria of |log2 FC| > 0.3 and FDR < 0.05 (Yu et al., 2012).

### Immune Infiltration Evaluation in GC

Estimation of STromal and Immune cells in MAlignant Tumor tissues using Expression data was used to evaluate the concentration of infiltrating non-tumor cells within the TME (Yoshihara et al., 2013). The proportions of infiltrating-stromal and immune cells in GC samples were quantified by stromal and immune scores using gene expression signatures.

Tumor Immune Estimation Resource (TIMER)^3^, is an open source server for comprehensive analysis of tumor-infiltrating immune cells across various cancers (Li et al., 2017). We explored the association between the expression of selected genes and the infiltration of six major immune cells in GC: CD4+ T cells, CD8+ T cells, B cells, neutrophils, macrophages, and dendritic cells. This same analysis was used to explore the correlation between hub genes and specific immune cell markers. The immune gene markers were selected based on the CellMarker online database^4^ and prior studies (Mlecnik et al., 2011; Tse and Kwong, 2015; Danaher et al., 2017).

CIBERSORT was used to predict the proportion of cells within the 22 human immune cell subsets in GC samples (Newman et al., 2015). The algorithm was suitable for microarray data analysis; therefore, we used the voom R package to correct mRNA expression values. CIBERSORT was run using the LM22 signature (downloaded from website https://cibersort.stanford.edu) and 1000 permutations, with output values of *P* < 0.05 preserved. The results were generated as a violin plot using the ggplot2 R package.

### LASSO and SVM-RFE Algorithms

Univariate cox analysis was used to identify potential hub genes with prognostic value. Then, we used LASSO and SVM-RFE algorithms to screen the genes with the best prognostic prediction value in GC. SVM-RFE and LASSO logistic regression was performed using the glmnet and e1071 packages, respectively (Friedman et al., 2010; Huang et al., 2014). LASSO regression was employed to minimize extra redundancy and irrelevance. SVM-RFE is a feature selection algorithm that ranks the features according to the recursive feature deletion sequence based on the support vector machine. Overlapping genes identified using these two algorithms were regard as hub genes.

### Verification of Predictive Performance and Differential Expression

The prognostic performance of the eight hub genes in GC patients was verified using the Kaplan–Meier plotter database^5^ (Lanczky et al., 2016). The Kaplan–Meier plotter can assess the significance of more than 50,000 genes for survival in nearly 1500 GC samples, based on the HGU133 Plus 2.0 array. The online web server Oncomine^6^ was used to examine hub gene expression levels across diverse cancer types and corresponding normal tissues. The threshold was set as *P*-value = 1E-4, fold change = 2, gene rank = 10%, data type = mRNA. We also selected GSE54129 and GSE79973 from the Gene Expression Omnibus (GEO) database^7^ to compare differences in hub gene expression between GC and normal gastric tissues.

### ssGSEA

Single-sample gene set enrichment analysis, a deconvolution algorithm based on gene set enrichment analysis (GSEA), transforms gene expression profiles into quantified immune cell fractions in single tumor samples. We used the GSVA R package to evaluate the proportion of 24 innate and adaptive immune cell subtypes in each GC sample. These cell types included natural killer (NK) cells, neutrophils, T effector memory (Tem), Tgd, mast cells, eosinophils, plasmacytoid dendritic cells (pDC), immature DCs (iDC), dendritic cells (DCs), macrophages, T follicular helper (TFH), T central memory cells (Tcm), Th2, Th17, CD56dim NK cells, regulatory T (Treg) cells, activated DCs (aDCs), cytotoxic cells, T cells, and B cells (Hanzelmann et al., 2013). The specific immune gene signatures were previously described (Bindea et al., 2013). We divided the immune cell infiltrating patterns of each sample into low-, median-, and high-infiltration groups using hierarchical agglomerative analyses based on Ward’s linkage and Euclidean distance. The relationship between the observed immune infiltrating patterns and overall survival in GC patients was also compared. Finally, we verified the correlation between hub gene expression and immune infiltrating levels in GC.

### Statistical Analysis

The statistical analysis was mainly performed in R version 3.5.1. Differences between groups were assessed using an independent *t*-test. Benjamini and Hochberg method was used for multiple correction test. Survival curves were generated using the GraphPad Prism 7 software and Kaplan–Meier plots databases. Survival analysis results were displayed as *P*-values and hazard ratios (HRs) from a log-rank test. Spearman’s correlation analysis was performed to evaluate the correlation between gene expression and immune infiltrating level. *P*-values < 0.05 were considered to indicate statistically significant results.

## Results

### Stromal Scores Identified as an Immune Indicator Associated With Prognosis in GC

**FIGURE 1:**
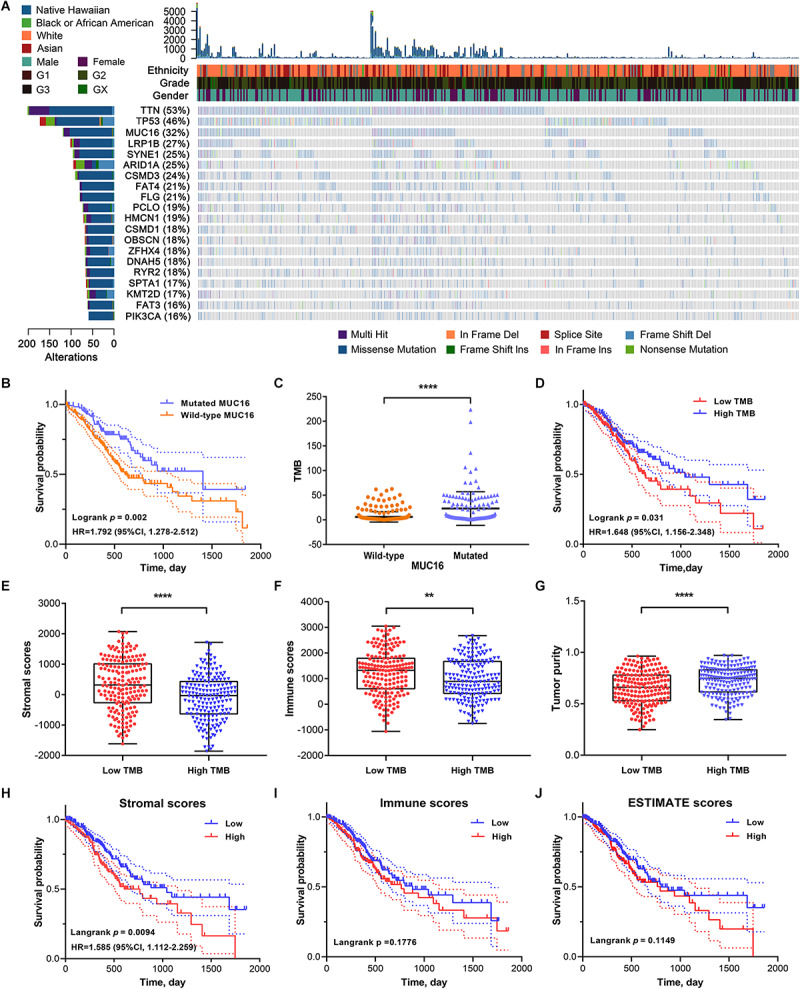
**(A)** The top twenty genes that were most frequently mutated in gastric cancer (GC) are displayed based on their mutation frequency in a waterfall plot. The corresponding gene mutation patterns and clinical status (ethnicity, grade, and gender) of the stomach adenocarcinoma (STAD) cohort are shown in the comment bar. **(B)** Kaplan–Meier survival analysis stratified by *MUC16* mutation status. **(C)** Association of tumor mutation burden (TMB) with *MUC16* mutation status. **(D)** Survival curves show that the low-TMB group had poorer overall survival (OS) than the high-TMB group. **(E)** Differences in stromal scores between low- and high-TMB groups. **(F)** Differences in immune scores between low- and high-TMB groups. **(G)** Differences in tumor purity between low- and high-TMB groups. **(H)** Kaplan–Meier survival analysis divided by stromal scores. **(I)** Kaplan–Meier survival analysis divided by immune scores. **(J)** Kaplan–Meier survival analysis divided by ESTIMATE scores. ****P < 0.0001; **P < 0.01.

Gastric adenocarcinoma mutation data were analyzed based on whole-exome sequencing. The 20 genes with the most significant mutations in GC samples were identified using the MutSigCV algorithm. The clinical profiles including ethnicity, tumor grade, and gender of the patients with GC that provided each sample are listed in Figure 1A. *MUC16* is one of the most significantly mutated genes in the GC cohorts. To explore its association with prognosis and TMB in patients with GC, we calculated the TMB for each GC sample. GC patients with *MUC16* mutations were significantly associated with a higher TMB (*P* < 0.001) and better survival outcomes (HR = 1.792, *P* = 0.002) than patients without *MUC16* mutations (Figures 1B,C). Based on the median value of TMB, we found that the high-TMB group was significantly associated with better survival prognosis than the low-TMB group (HR = 1.648, *P* = 0.031) in GC (Figure 1D).

To investigate the correlation between the TME and TMB in GC, we calculated stromal and immune scores, and tumor purity of each sample using the ESTIMATE algorithm. GC cohorts were divided into two groups based on the median value of each index. The low-TMB group tended to have higher stromal and immune scores but lower tumor purity (*P* < 0.05), indicative of a negative correlation between stromal scores and TMB (Figures 1E–G). In Kaplan–Meier survival analysis, the high stromal scores group had significantly poorer prognosis (HR = 1.585, *P* = 0.009) than did the low stromal group in GC (Figure 1H). The survival outcome was not statistically significant in the different immune (*P* = 0.178) and ESTIMATE (*P* = 0.115) scores groups (Figures 1I,J). These results suggest that TMB is negatively correlated with stromal scores, and the underlying molecular mechanisms in GC warrant further investigation.

### Identification of Stromal Scores Relevant Gene Modules Using WGCNA

**FIGURE 2:**
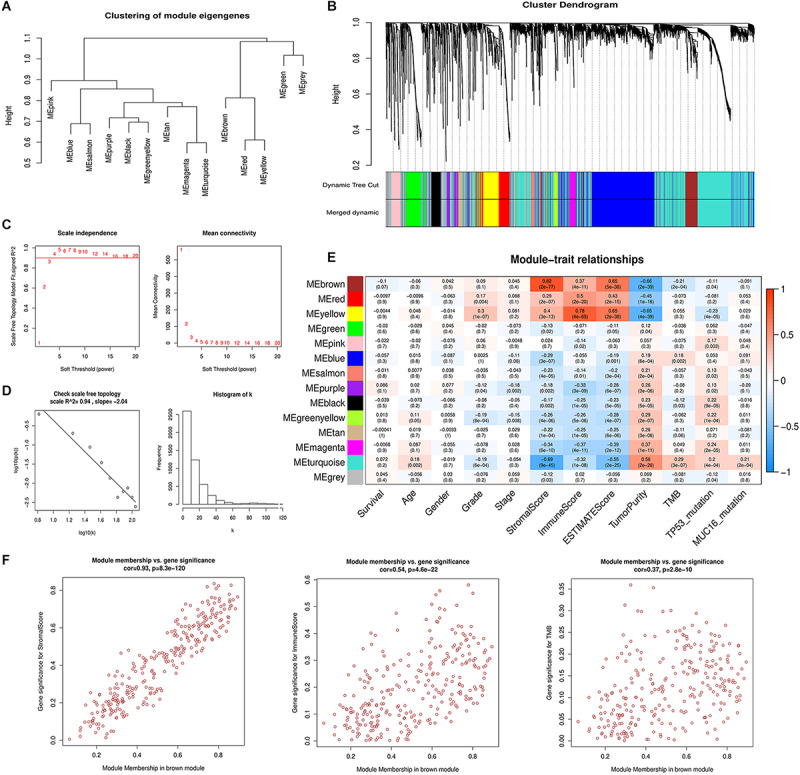
Weighted gene co-expression network analysis. **(A)** Hierarchical clustering of 14 module eigengenes **(B)** The gene cluster dendrogram is generated according to the dissimilarity measure. Each branch of the cluster dendrogram represents a gene and corresponds to 14 different co-expression modules. **(C)** The scale-free fit index and mean connectivity are calculated with different soft-thresholding powers (β) from 1 to 20. **(D)** The co-expression network connectivity distribution is demonstrated in the plot. The soft threshold β is selected as 4. The logarithm of the network connectivity *k* is shown on the *x*-axis, while the logarithm of the corresponding frequency distribution is shown on the *y*-axis. The distribution follows a straight line, representing an approximately satisfactory scale-free topology network (correlation coefficient = 0.94). **(E)** The correlation between different module eigengenes and various clinical parameters of gastric cancer in a heatmap. The brown module is most positively associated with stromal scores and the yellow module is most positively associated with immune scores. **(F)** Scatter plot showing the brown module eigengenes.

To identify gene modules with the most significant immunological features and to elucidate the mechanisms underlying the role of the TME in GC development and prognosis, we constructed a weighted gene co-expression network. After eliminating 23 outlier samples (Supplementary Figure S2A), genes with the highest 75% variance were placed in a cluster dendrogram. To satisfy a scale-free network (*R*^2^ = 0.94), the soft threshold β was set at 4 and the topology model fit index was set at 0.9 (Figures 2C,D). After identifying the eigengenes of each module, 14 merged modules were obtained (Figures 2A,B).

The correlation between the modules and clinical phenotypes including survival status, age, gender, histologic grade, pathologic stage, stromal scores, immune scores, ESTIMATE scores, tumor purity, TMB, *TP53* mutation status, and *MUC16* mutation status were calculated and shown as a heatmap based on MMs and GS (Figure 2E and Supplementary Figure S2B). The brown and yellow modules strongly correlated with tumor immune-microenvironment-related phenotypes, and contained 273 and 272 genes, respectively. The brown module was most significantly associated with stromal score (Cor = 0.82, *P* = 2e-77) and its correlation coefficients with immune scores and tumor purity were 0.37 and −0.66, respectively (*P* < 0.001). Additionally, the brown module was negatively correlated with TMB (Cor = −0.21, *P* = 2e-4). The yellow module correlated with immune score (Cor = 0.78, *P* = 4e-65). Our previous analyses suggested a significant prognosis difference between different stromal scores and TMB groups. Therefore, we chose the brown module as the module of interest for further analysis. We performed correlation analysis between MM of the brown module and GS of different clinical phenotypes (Figure 2F). The correlation between MM and stromal score GS showed a favorable linear fitting (Cor = 0.93, *P* = 8.3e-120).

### Functional Enrichment Analysis for the Module of Interest

**FIGURE 3:**
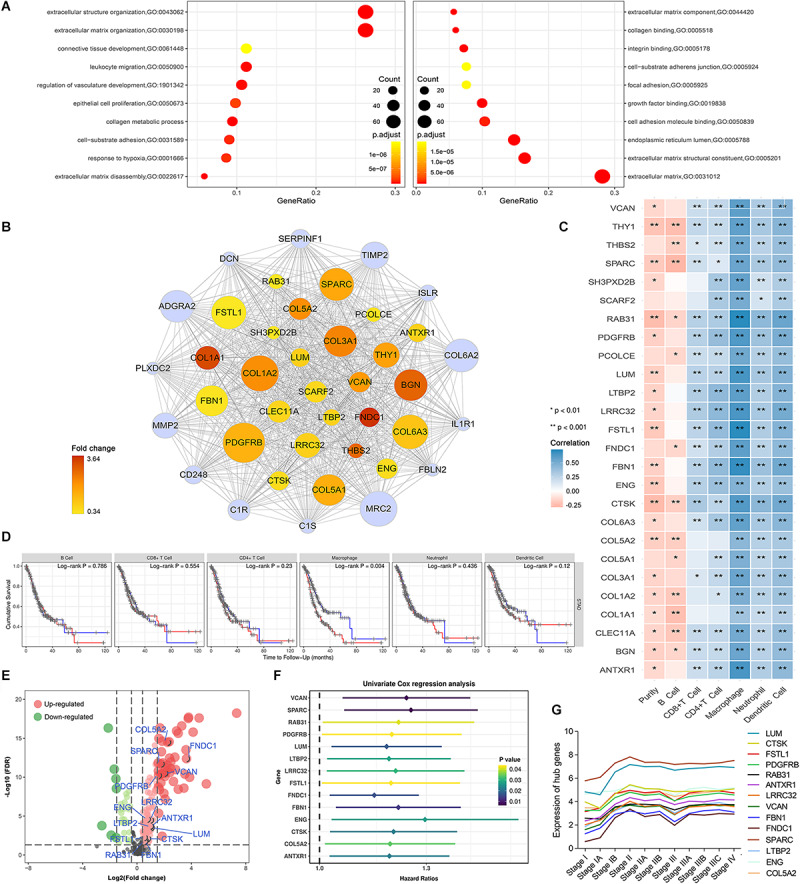
Identification of potential hub genes and their correlation with immune infiltration in gastric cancer. **(A)** Brown module gene ontology enrichment analysis. **(B)** The 40 genes with the highest intramodular connectivity value within the brown module are demonstrated. Node size is based on the intramodular connectivity within the brown module. The node color changes from yellow to red in ascending order according to the foldchange of the potential hub genes in this study. **(C)** Correlation of potential hub gene expression with immune infiltrating level in stomach adenocarcinoma based on TIMER database; blue and red indicate positive and negative correlations, respectively. **(D)** Correlation of infiltrating levels of various immune cells with overall survival in stomach adenocarcinoma according to the TIMER database. Red line represents high infiltrating levels (top 50%), blue line represents low infiltrating levels (bottom 50%). **(E)** Volcano plot showing the differentially expressed genes in the brown module. **(F)** Univariate Cox analysis for potential hub genes obtained 14 genes associated with prognosis in gastric cancer. **(G)** Trendline of the expression of potential hub genes at various gastric cancer stages.

GO analysis was used to reveal the underlying biological mechanisms in the brown module using the “clusterProfile” R package. Consistent with WGCNA results, functional annotation clustering of brown module genes exhibited strong association with stroma-related biological functions, including “extracellular structure organization,” “extracellular matrix disassembly,” “integrin binding,” “extracellular matrix organization,” “cell-substrate adherens junction,” and “collagen metabolic process.” Important biological functions including “leukocyte migration,” “regulation of vasculature development,” and “response to hypoxia” were also significantly enriched in the brown module (Figure 3A).

### Screening of Differentially Expressed Potential Hub Genes

We performed differential expression analysis for genes in the brown module to identify potential tumorigenic factors. Owing to the biological cascading effect, potential hub genes with low expression values within tumors might play important roles in TME development. To eliminate such bias, we adopted a relatively broad inclusion criterion. With the cut-off threshold set as |log 2 FC| > 0.3 and FDR < 0.05, 160 differentially expressed genes DEGs in the brown module were screened out. Of these DEGs, 129 were up-regulated, and 31 were down-regulated in GC tissues (Figure 3E). To identify the most centrally connected genes within the weighted gene co-expression network, we screened out 40 brown module genes with the highest intramodular connectivity. Among them, 26 genes were significantly up-regulated and defined as potential hub genes (Figure 3B).

### Stromal-Relevant Hub Genes Are Associated With Macrophages Infiltration in GC

Based on the brown module’s dual positive association with stromal and immune scores, we postulated that interplay between immune and stromal cells has important roles in carcinogenesis. We used the TIMER database to investigate the correlation between the 26 stromal-related DEGs and tumor immune infiltrates. The potential hub genes and their correlation with infiltrating immune cell concentration within the TME was assessed (Figure 3C). Notably, High expression of these genes was moderately to strongly positively correlated with high macrophage infiltration levels (Cor = 0.4–0.8, *P* < 0.05).

We assessed the prognostic significance of the infiltrating levels of the six immune cells in GC using the TIMER survival module. We found that high macrophage infiltration levels were associated with a poorer prognosis in GC (Figure 3D*missing*, P = 0.004). These results led to the hypothesis that stromal-related hub genes might promote tumor development by regulating macrophage infiltration in GC.

### Preliminary Identification of Prognostic Hub Genes Using the Machine Learning Method

**FIGURE 4:**
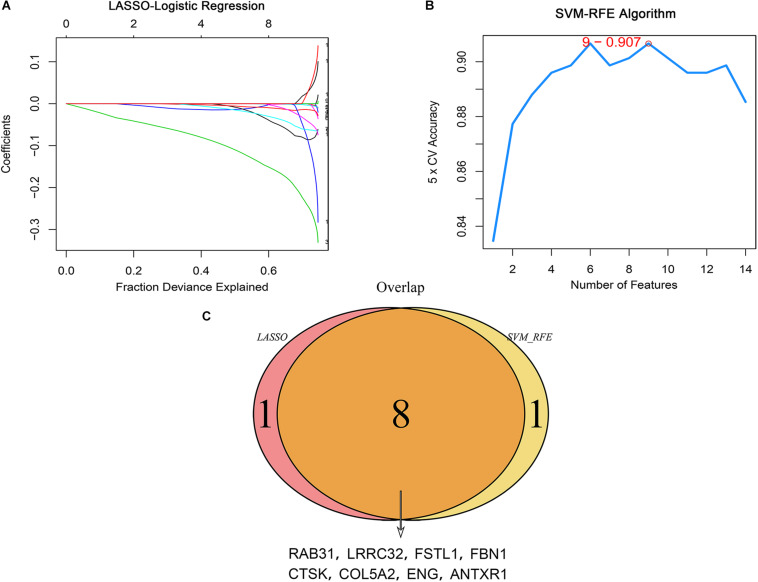
Identification of hub genes associated with prognosis using the machine learning method. **(A)** LASSO coefficient profiles of candidate hub genes. Each curve corresponds to a candidate gene. **(B)** The SVM-RFE algorithm identified candidate genes with the best predictive performance and lowest error bar (5 × CV accuracy = 9–0.907). **(C)** Venn plot showing hub genes identified in common by the two algorithms.

**FIGURE 5:**
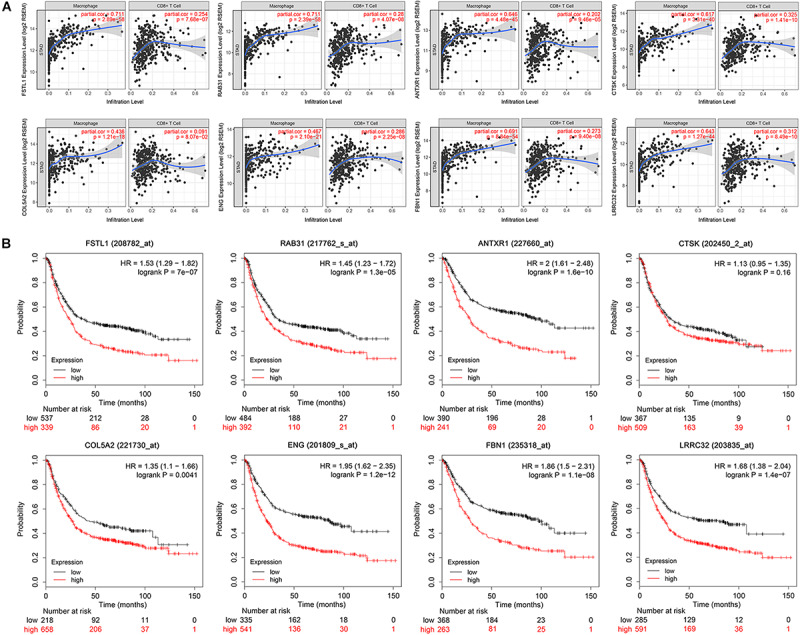
Immune correlation and survival analysis of the eight hub genes. **(A)** Scatter plot showing a strong and positive correlation (Cor = 0.4–0.8, *P* < 0.05) between the expression level of eight selected hub genes and the macrophage infiltration level. CD8+T set as control. **(B)** Survival analysis validation of the eight hub genes in gastric cancer using Kaplan–Meier plotter.

We performed univariate Cox regression analysis on 26 potential hub genes and identified 14 genes significantly associated with unfavorable prognosis in GC (Figure 3F). We mapped their expression trends based on clinical GC stages and found significant differences in expression between stage I and the other stages (Figure 3G). These results suggest that the potential hub genes play important roles in early tumor development stages. The LASSO and SVM-RFE algorithms identified nine characteristic genes respectively (Figures 4A,B). By overlapping the biomarkers from the two algorithms, we identified eight hub genes with the best prognostic predictive performance (Figure 4C). The co-expression relationship between these eight hub genes is shown in Supplementary Figure S3. These eight prognostic hub genes, which are more strongly related to macrophage infiltration than the other potential hub genes (Figure 5A), are as follows: Ras-related protein 31 (*RAB31*, Cor = 0.711, *P* = 2.39e-58); Follistatin like 1 (*FSTL1*, Cor = 0.711, *P* = 2.89e-58); Fibrillin 1 (*FBN1*, Cor = 0.691, *P* = 8.84e-54); Anthrax toxin receptor 1 (*ANTXR1*, Cor = 0.646, *P* = 4.48e-45); Leucine rich repeat containing 32 (*LRRC32*, Cor = 0.643, *P* = 1.27e-44); Cathepsin K (*CTSK*, Cor = 0.617, *P* = 3.31e-40); Collagen type V alpha 2 chain (*COL5A2*, Cor = 0.436, *P* = 1.21e-18); and Endoglin (*ENG*, Cor = 0.467, *P* = 2.25e-8).

### Survival Analysis and Gene Expression Validation

**FIGURE 6:**
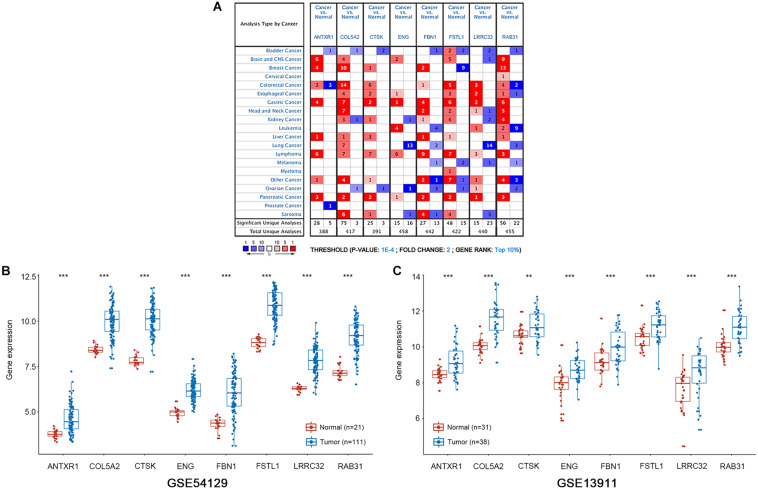
mRNA expression patterns of the eight hub genes was verified with the Oncomine and GEO databases. **(A)** The Oncomine database shows the mRNA expression differences between various cancers and corresponding normal tissues. The threshold is shown at the bottom. The figure in the colored cell indicates the number of data sets satisfying the threshold. The red cells show that hub genes are overexpressed in tumor tissues, while the blue cells show that hub genes are downregulated in tumor tissues. **(B,C)** Boxplots show the expression of eight hub genes in gastric cancer and normal gastric tissues from GSE54129 and GSE13911 datasets. ***P < 0.001; **P < 0.01.

We conducted survival validation on the eight hub genes using the Kaplan–Meier plotter database. Seven of the hub genes with high expression (*FSTL1, RAB31, ANTXR1, COL5A2, ENG, FBN1*, and *LRRC32*) were found to predict poorer overall survival than their low-expression counterparts (*P* < 0.05) (Figure 5B). We verified hub gene mRNA expression levels across different types of cancers in the Oncomine database. These hub genes are overexpressed in GC and in other cancers including pancreatic cancer and lymphoma (Figure 6A). The eight hub genes were all ranked at the highest 1% in GENE RANK. Five genes had four or more datasets supporting their aberrant expression in GC with the threshold of fold change = 2, *P*-value = 1E-4. Moreover, we used datasets GSE54129 and GSE79973 from GEO database to further examine hub gene expression differences in GC and normal tissues (Figures 6B,C). The results show that these hub genes are more highly expressed in GC than in normal gastric tissues (*P* < 0.05).

### Revalidation of the Correlation Between Hub Genes and Tumor Immune Characterization

**FIGURE 7:**
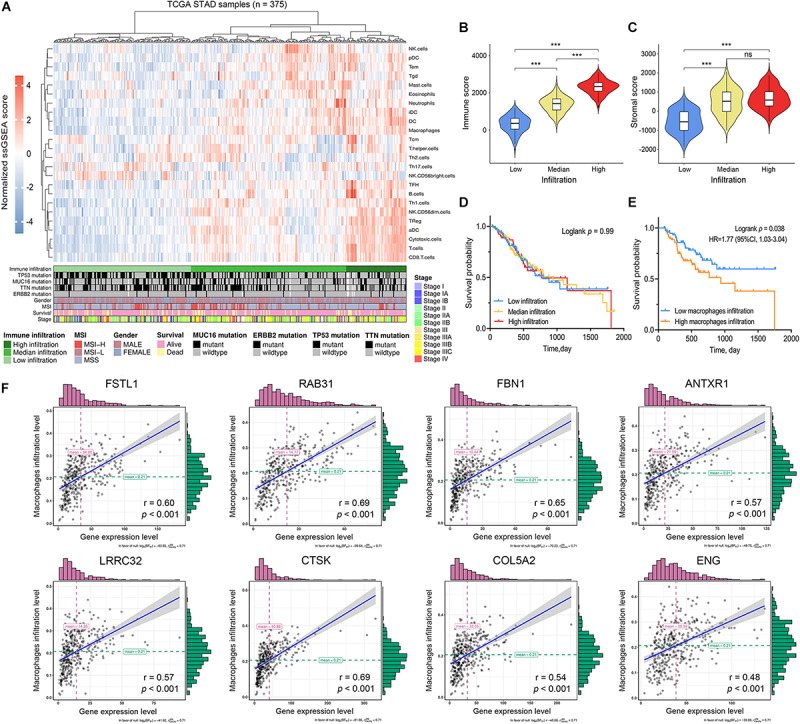
Immune characterization of gastric cancer. **(A)** Gastric cancer samples (*n* = 375) from the TCGA cohort were divided into low immune infiltration, median immune infiltration, and high immune infiltration with unsupervised clustering using single sample gene-set enrichment analysis (ssGSEA) scores based on 24 immune signatures. **(B)** The relationship between immune scores and immune infiltration clusters. **(C)** The relationship between stromal scores and immune infiltration clusters. **(D)** Kaplan–Meier survival analysis divided by immune infiltration clusters **(E)** Kaplan–Meier survival analysis divided by macrophage infiltration level. **(F)** The correlation between hub gene expression levels and macrophage infiltration levels based on ssGSEA scores. ***P < 0.001; ns: no significance.

To validate the relationships between the hub genes with tumor immune characteristics we used ssGSEA to reevaluate the immune infiltration signature of each sample in 24 immune cell types. The 375 GC samples were distinguished as having high, medium, and low immune infiltration patterns with the corresponding clinical characteristics (Figure 7A). We compared the stromal and immune scores between the three immune infiltration patterns (Figures 7B,C). We found that high immune-infiltration groups have higher immune scores than do the low- and medium immune infiltration groups (*P* < 0.001). Although the difference in stromal scores between high and medium infiltration groups was not significant, the median value of the stromal scores was higher in the high-infiltration group (*P* < 0.001). We compared survival differences between the three groups and found no significant differences (Figure 7D). Survival significance differences was observed when we divided the groups into high- and low macrophage infiltration levels using upper and lower quartiles (Figure 7E, HR = 1.77, *P* = 0.038). Based on the results of ssGSEA analysis, we further assessed the correlation between hub gene expression and the infiltrating levels of 24 kinds of immune cells (Supplementary Figure S4). The high expression level of the selected hub genes was significantly associated with infiltration of various immune cells, especially macrophages, in GC (Figure 7F).

### Exploration of the Correlation Between Hub Genes and Macrophage-Related Immune Markers

**FIGURE 8:**
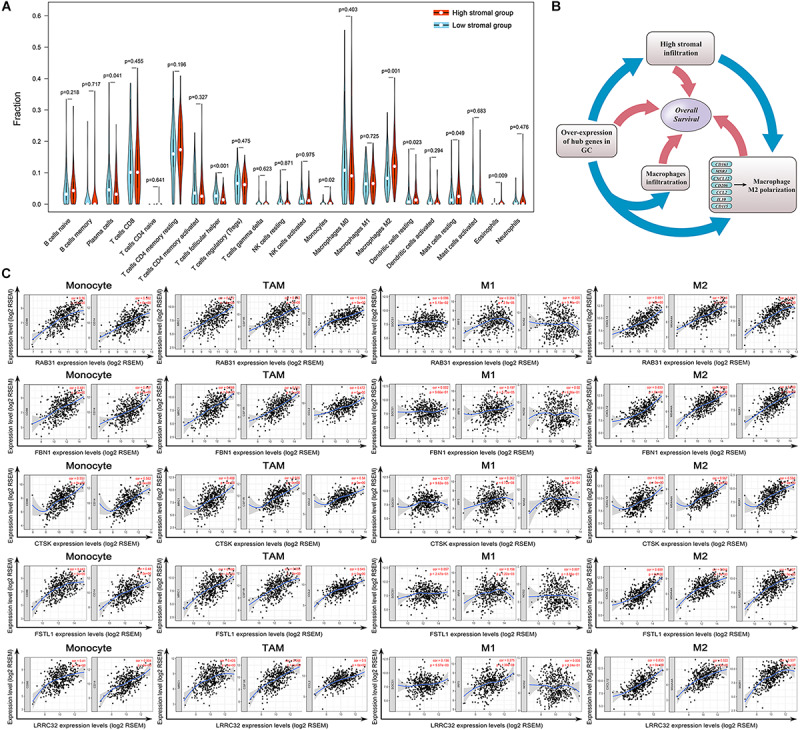
Evaluation of the stromal-related hub genes with infiltrating macrophage subtypes. **(A)** The difference in immune cell infiltration levels between high- and low-stroma groups. **(B)** Scatterplot showing the correlations between hub gene expression and markers of monocytes, tumor-associated macrophages (TAMs), and M1 and M2 macrophages. Monocytes immune markers include CD86 and CD14. TAM immune markers include MRC1, CSF1R, and CCL2. M1 macrophage immune markers include SOCS1, IRF5, and NOS2. M2 macrophage immune markers include CD163, MS4A4A, and MSR1. **(C)** Overexpression of hub genes is positively correlated with high stromal infiltration, high macrophage infiltration, and macrophage polarization in gastric cancer. Factors associated with poor prognosis in gastric cancer are indicated with rectangles.

We used CIBERSORT to evaluate the differences in immune infiltration between high- and low stromal groups in GC to elucidate the underlying connection between tumor stroma and infiltrating-immune cells within the TME. With the cut-off standard set as *P*-value < 0.05, we obtained 161 and 83 samples in the high- and low stromal groups, respectively. The high-stromal group had higher infiltration of monocytes (*P* = 0.020), M2 macrophages (*P* = 0.001), resting mast cells (*P* = 0.049), and resting DCs (*P* = 0.023) than the low-stromal group (Figure 8A). This highlights the correlation between tumor stroma and tumor-associated macrophages (TAMs). It is feasible that a close interaction exists between stromal-related hub genes and TAMs in tumor progression. Therefore, we analyzed the correlation between hub gene expression and specific gene markers of macrophages in various immune cell subtypes; these included CD86, CD14, CD16 of monocytes; CCL2, CD115, CD206, and IL10 of TAMs; INOS, IRF5, SOCS1, CCR7, TSPO, ROS, IL6, and CXCL10 of M1 macrophages; and CXCL12, MS4A4A, MAR1, CD36, VISIG4, DCIR, CD184, and CD163 of M2 macrophages. Interestingly, eight hub genes were all significantly correlated with the gene markers of TAMs, M2 macrophages, and monocytes (*P* < 0.01, Table 1). There was a moderate to strong correlation between five hub genes (*RAB31*, *FBN1*, *CTSK*, *FSTL1*, *LRRC32*) and CD86 and CD14 of monocytes; MRC1, CSF1R, and CCL2 of TAMs; and CXCL12, MS4A4A, and MSR1 of M2 macrophages. Furthermore, weak correlation between the hub genes and SOCS1, IRF5 and NOS2 of M1 macrophages was observed (Figure 8B). These findings suggest that hub genes might regulate macrophage polarization in GC.

**TABLE 1:** Correlation analysis of the eight hub genes with immune markers of different macrophage phenotypes in TIMER.

Description	Markers	RAB31		FBN1		CTSK		LRRC32		FSTL1		ANTXR1		ENG		COL5A2	
		Cor	*P*	Cor	*P*	Cor	*P*	Cor	*P*	Cor	*P*	Cor	*P*	Cor	*P*	Cor	*P*
Monocyte	CD86	0.560	***	0.481	***	0.551	***	0.410	***	0.443	***	0.403	***	0.307	***	0.382	***
	CD14	0.582	***	0.497	***	0.582	***	0.504	***	0.480	***	0.457	***	0.438	***	0.424	***
	CD16	0.568	***	0.514	***	0.516	***	0.426	***	0.425	***	0.457	***	0.167	***	0.490	***
TAM	CCL2	0.544	***	0.472	***	0.540	***	0.500	***	0.545	***	0.458	***	0.489	***	0.367	***
	CD115	0.572	***	0.621	***	0.555	***	0.566	***	0.555	***	0.476	***	0.479	***	0.381	***
	CD206	0.572	***	0.566	***	0.488	***	0.425	***	0.498	***	0.449	***	0.327	***	0.419	***
	IL10	0.520	***	0.500	***	0.506	***	0.449	***	0.446	***	0.413	***	0.378	***	0.380	***
M1	INOS	–0.005	na	0.020	na	0.054	na	0.005	***	0.007	na	–0.051	na	–0.018	na	0.098	*
Macrophage	IRF5	0.204	***	0.197	***	0.262	***	0.275	***	0.158	**	0.182	***	0.262	***	0.091	na
	SOCS1	0.096	na	0.002	na	0.127	**	0.136	**	0.057	na	–0.049	na	0.318	***	0.012	na
	CCR7	0.337	***	0.352	***	0.316	***	0.405	***	0.362	***	0.183	***	0.510	***	0.066	na
	TSPO	–0.235	***	–0.302	***	–0.119	*	–0.197	***	–0.334	***	–0.226	***	–0.111	*	–0.155	**
	ROS1	–0.005	na	0.030	na	0.059	na	0.007	na	0.041	na	0.085	na	0.054	na	0.108	*
	IL6	0.364	***	0.302	***	0.326	***	0.249	***	0.331	***	0.285	***	0.315	***	0.381	***
	CXCL10	0.253	***	0.161	**	0.223	***	0.120	*	0.122	na	0.109	*	–0.013	na	0.173	***
M2	CXCL12	0.601	***	0.633	***	0.508	***	0.633	***	0.659	***	0.471	***	0.714	***	0.377	***
Macrophage	MS4A4A	0.614	***	0.583	***	0.567	***	0.522	***	0.544	***	0.496	***	0.340	***	0.383	***
	MSR1	0.637	***	0.603	***	0.584	***	0.537	***	0.537	***	0.540	***	0.302	***	0.490	***
	CD36	0.468	***	0.558	***	0.409	***	0.515	***	0.558	***	0.395	***	0.508	***	0.286	***
	VSIG4	0.634	***	0.568	***	0.560	***	0.495	***	0.530	***	0.505	***	0.326	***	0.447	***
	DCIR	0.472	***	0.431	***	0.480	***	0.317	***	0.399	***	0.331	***	0.312	***	0.333	***
	CD184	0.462	***	0.476	***	0.466	***	0.479	***	0.485	***	0.404	***	0.417	***	0.245	***
	CD163	0.578	***	0.590	***	0.488	***	0.460	***	0.492	***	0.454	***	0.332	***	0.438	***

TAM, tumor-associated macrophage; Cor, R value of Spearman’s correlation; *P < 0.05; ** *P* < 0.01; *** *P* < 0.001; na *P* > 0.05.

The present investigation of TME in GC indicates a complex relationship between the genome and immune infiltration. Overexpressed prognostic hub genes positively correlate with high stromal infiltration and may also participate in macrophage recruitment and M2 macrophage polarization. High-stromal-group patients were also observed to have higher M2 macrophage infiltration. The adverse prognostic value of each factor was verified independently in survival analysis (Figure 8C).

## Discussion

Biological behaviors of cancers are determined by genetic instability (such as TMB), cancer cell epigenetic abnormalities, and the surrounding milieu (such as TME) that the cancer cells interact with for growth, survival, proliferation, and metastasis (Hanahan and Weinberg Robert, 2011; Mlecnik et al., 2011). Infiltrating stromal and immune cells are major components of normal cells within tumor tissue (Yoshihara et al., 2013). To elucidate the components of TME in GC, we utilized the ESTIMATE algorithm to evaluate the immune phenotypes of GC with immune and stromal scores. Our results showed that stromal scores, but not immune scores, were significantly correlated with survival outcomes in GC, indicating that the stromal compartment plays an important role in GC. Previous studies have indicated that stromal cells, like fibroblasts, are much more crucial in TME formation in GC than inflammatory cells (Komohara and Takeya, 2017; Ham et al., 2019; Kobayashi et al., 2019).

Tumor mutational burden, a novel biomarker of immunotherapy response, is based on the notion that mutation-associated neoantigens can activate immune cells to eliminate cancer cells: the higher the TMB, the better the therapeutic effect (Samstein et al., 2019). Here, the low-TMB group showed significantly poorer survival compared with high-TMB group. In addition, the low-TMB group tended to have higher stromal scores in GC. Given the validity of TMB as a biomarker for immunotherapy response, we speculated that the population that was not susceptible to immunotherapies (i.e., the low-TMB group) are highly associated with stromal cells (high stromal score) within the TME, which is correlated with unfavorable prognosis.

To elucidate the potential connection between the genome and TME in GC, we systematically clustered the co-expressed genes by WGCNA analysis. This approach allowed us to identify gene modules most related to cancer immunological phenotypes, especially stromal scores and TMB. The brown module showed strong positive correlation with stromal scores, moderate correlation with immune scores, and negative correlation with TMB (Figure 2E). Functional enrichment analysis for the brown module also confirmed its strong correlation with stromal-related phenotypes, including ECM organization and leukocyte migration. This suggests that special genomic abnormalities may facilitate malignant growth by affecting stromal structure or the immunogenicity of the TME in GC. Therefore, we performed differential expression analysis for brown module genes with the highest intramodular connectivity in order to identify potential oncogenes. We adopted a combination strategy, integrating three different algorithms, to screen the prognostic biomarkers from DEGs. Based on univariate Cox analysis determining the prognostic DEGs, LASSO, and SVM-RFE methods were used to identify biomarkers with distinct characteristics. After survival analysis and expression validation, eight brown module hub genes were identified as adverse prognostic factors in GC. Growing evidence suggests that the stromal compartment can influence antitumor immune responses and regulate tumor immunology (Komohara and Takeya, 2017). We investigated whether stromal-related hub genes play an immunomodulatory role within the TME. To this end, we used TIMER and ssGSEA to analyze the relationship between hub gene expression and tumor immune infiltration in GC. The results show a strong positive correlation between hub gene expression and macrophage infiltration in GC. The similarity of the immune correlation analysis results using two independent methodologies indicates the robustness of the results. We additionally observed that high macrophage infiltration levels represent as an adverse prognostic factor in GC (Figures 3D, 7E). To reveal the mechanisms underlying the relationship between stromal score and prognosis, we compared the immune-infiltrating cell fractions of each sample between high and low stromal groups. Intriguingly, the high-stromal group was found to have higher proportions of monocytes and M2 macrophages. Accordingly, further analysis was performed to explore the correlation between stromal-related hub genes and different immunophenotypes of macrophages in order to elucidate the role of hub genes in regulating tumor immunology in GC. Immune markers of monocytes and TAMs, such as CD86 and CSF1R, showed a strong positive correlation with hub gene expression, suggesting that the stromal-related hub genes promote the recruitment of circulating monocytes into the TME and facilitate their differentiation into TAMs. Moreover, M1 macrophage immune markers, such as SOCS1 and NOS2, demonstrated weak or even negative correlation with the expression of hub genes, while M2 macrophage immune markers, such as CXCL12, MSR1, and MS4A4A, exhibited moderate to strong correlation. These results demonstrate the underlying role of hub genes in regulating the recruitment of macrophages and polarization of TAMs in GC.

Evidence indicates that stromal cells may shape an immunosuppressive microenvironment by secreting cytokines and promoting M2 polarization of macrophages (Comito et al., 2014). Notably, Cathepsin K (CTSK), a lysosomal cysteine protease of the peptide protein C1 family, is implicated in matrix remodeling and angiogenesis (Krieg and Lipford, 2008). CTSK overexpression has been detected in breast, prostate, and gastrointestinal cancers, and correlated with tumor stroma (Kleer et al., 2008; Li R. et al., 2019). CTSK was identified as a metastatic-related protein regulated by gut microbiota in colorectal cancers, and its effect on M2 polarization of TAMs has been confirmed (Li R. et al., 2019). The evidence correlating stromal-related hub genes with M2 macrophage polarization support further exploration of the potential of these genes as immunotherapeutic targets.

Of the eight stromal-related hub genes, *FSTL1*, *FBN1*, *COL5A2*, and *LRRC32* encode extracellular proteoglycans, glycoproteins, and other components within the ECM, playing a fundamental role in determining ECM composition (Andrikopoulos et al., 1995; Dubuisson et al., 2001; Carrillo-Galvez et al., 2015; Mattiotti et al., 2018). ANTXR1, Rab31, and ENG are transmembrane or membrane proteins engaged in signal transduction and transmembrane trafficking (Chen et al., 2013; Carrillo-Galvez et al., 2015). FSTL1 is involved in multiple tumor biological processes, including metastasis and regulation of the immune response. It is noteworthy that FSTL1 is significantly related to tumor metastasis, and up or down regulation of FSTL1 in different cancers greatly induces their migratory and invasive capacity, leading to tumor dissemination (Kudo-Saito et al., 2013; Ni et al., 2018; Chiou et al., 2019). FSTL1 exerts this effect on migration through the reduction of various cytokines, including metalloproteinase-2 (MMP-2), CCL2, and CXCL12 (Chan et al., 2009). Moreover, FSTL1 is considered a determinant of immune dysfunction mediated by mesenchymal stroma/stem cells (MSCs) and immunoregulatory cells, and may therefore represent a target to suppress cancer progression (Kudo-Saito et al., 2018).

Rab31 is a member of the RAB family derived from monomeric GTP-binding proteins, which regulate intracellular transportation (Cheng et al., 2005). Numerous studies have reported that Rab31 participates in tumor initiation and progression across various cancers, including breast cancer, glioblastoma, and pancreatic cancer (Ioannou et al., 2015; Pan et al., 2016; Li H. et al., 2019). Rab31 was found to play critical role in tumor development and is an independent prognostic factor in breast cancer (Kotzsch et al., 2017). Recently, Rab31 was recognized to function as an oncogene in GC tumorigenesis and progression by interacting with GLI1, which represents a therapeutic target in GC (Tang et al., 2018). ANTXR1, also known as tumor endothelial marker 8 (TEM8), is widely considered a potential target for cancer therapy because of its effect on tumor angiogenesis. Consistent with our results, ANTXR1 is widely expressed on tumor-associated perivascular stromal cells, which strongly promotes angiogenesis within the TME (Bagley et al., 2009). Recent studies have revealed that antibodies targeting ANTXR1 exert unique antitumor effects by selectively inhibiting stromal endothelial cells (Liu et al., 2016). In addition, ANTXR1 is regarded as a potential target for CAR T cell immunotherapies in gastric adenocarcinoma (Sotoudeh et al., 2019), which makes it more applicable for clinical treatment.

Using integrated bioinformatic and machine learning algorithms, we elucidated the genomic landscape of GC and its correlation with TMB, the stromal compartment, and immune cell infiltration. Eight stromal-related prognostic hub genes were found to play pleiotropic roles in the TME of GC. These hub genes inhibit the formation of an immunosuppressive microenvironment, therefore representing potential therapeutic targets. The present findings were based on retrospective datasets, and biological experiments are needed to verify our results.

## Data Availability Statement

The datasets generated for this study are available on request to the corresponding author.

## Author Contributions

WZ and XC: conceptualization and research supervision. SW, JLu, JLo, CS, SM, YS, and JN: experiments. SW, JLu, and JLo: data analysis. JLu and SW: original draft writing. SW, JLu, and WZ: review, editing, and final approval. All authors contributed to the article and approved the submitted version.

## Conflict of Interest

The authors declare that the research was conducted in the absence of any commercial or financial relationships that could be construed as a potential conflict of interest.

## Footnotes

1 https://portal.gdc.cancer.gov/

2 http://asia.ensembl.org/index.html

3 http://timer.cistrome.org/

4 http://biocc.hrbmu.edu.cn/CellMarker/

5 http://kmplot.com/analysis

6 https://www.oncomine.org

7 https://www.ncbi.nlm.nih.gov/geo/

## Supplementary Material

FIGURE S1 Work-flow of the bioinformatics analysis procedure.

FIGURE S2 Sample clustering dendrogram. **(A)** Sample clustering was conducted to detect outlier samples. Outlier samples (*n* = 23) were eliminated. **(B)** Trait heatmap and sample dendrogram after outlier sample elimination. A total of 14 samples were included in the dendrogram. Color concentration is in proportion to survival, age, gender, stage, stromal score, immune score, ESTIMATE score, tumor purity, tumor mutation burden, *TP53* mutation status, and *MUC16* mutation status.

FIGURE S3 Correlation analysis of the eight hub genes with 24 immune signatures in ssGSEA.

FIGURE S4 Co-expression analysis of the hub genes at transcriptional level.

TABLE S1 Clinical features of STAD patients in TCGA database.

## Abbreviations

CIOBSORT: Cell-type Identification By Estimating Relative Subsets Of RNA Transcripts
DEGs: differentially expressed genes
ECM: extracellular matrix
ESTIMATE: Estimation of STromal and Immune cells in MAlignant Tumor tissues using Expression data
GC: gastric cancer
GS: gene significance
HR: hazard ratio
LASSO: least absolute shrinkage and selection operator
MEs: Module Eigengens
ssGSEA: single-sample gene set enrichment analysis
SVM-RFE: support vector machine-recursive feature elimination
TAMs: tumor-associated macrophages
TCGA: The Cancer Genome Atlas
TIMER: Tumor Immune Estimation Resource
TME: tumor microenvironment
WGCNA: weighted gene co-expression network analysis.
