# Supplementary figures and images for: Gastric Cancer Tumor Microenvironment Characterization Reveals Stromal-Related Gene Signatures Associated With Macrophage Infiltration
Source: Front Genet. 2020 Jun 30;11:663. doi: 10.3389/fgene.2020.00663 (PMC7339942; doi:10.3389/fgene.2020.00663)

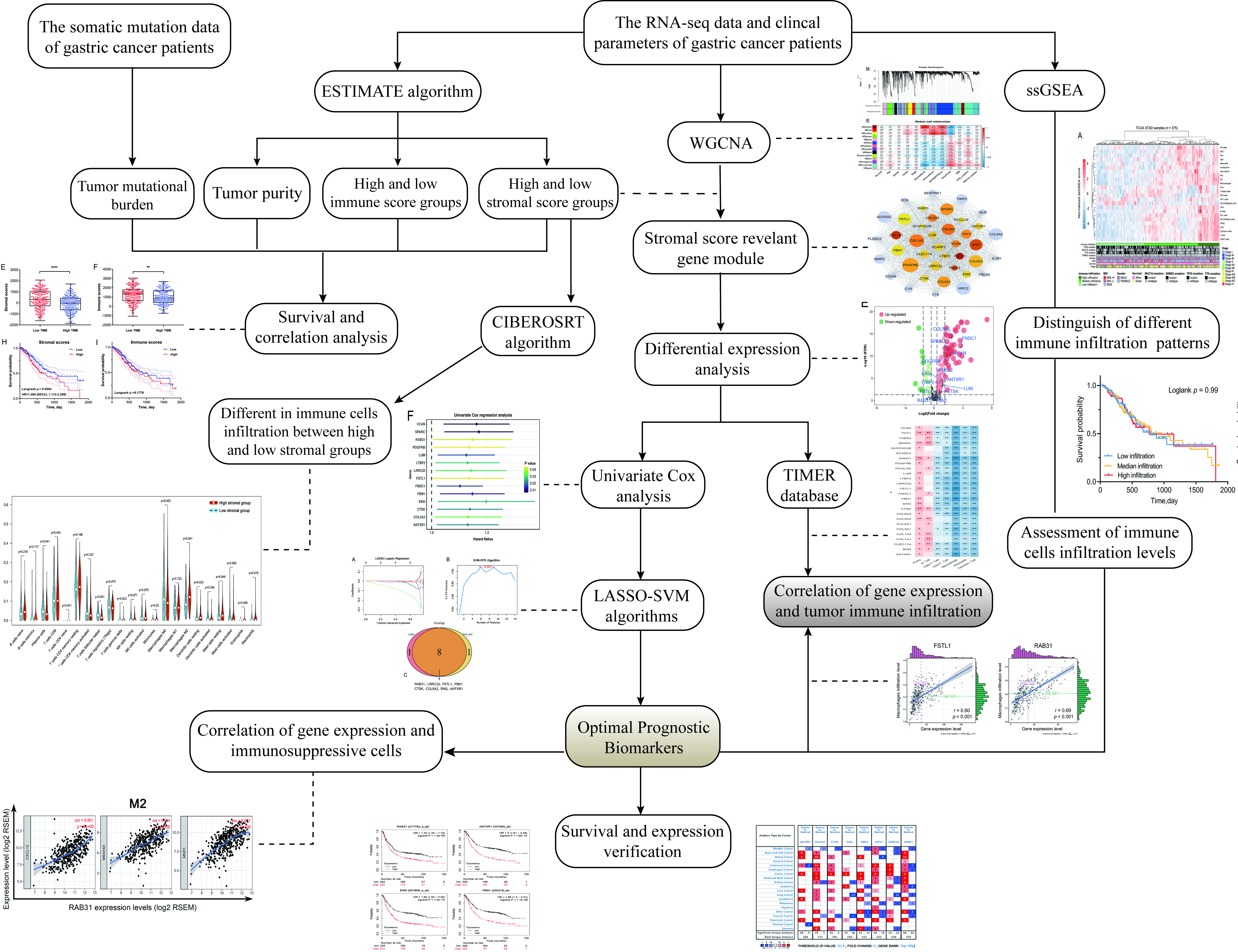

Supplement: FIGURE S1 — Work-flow of the bioinformatics analysis procedure. [file Image_1.TIF]

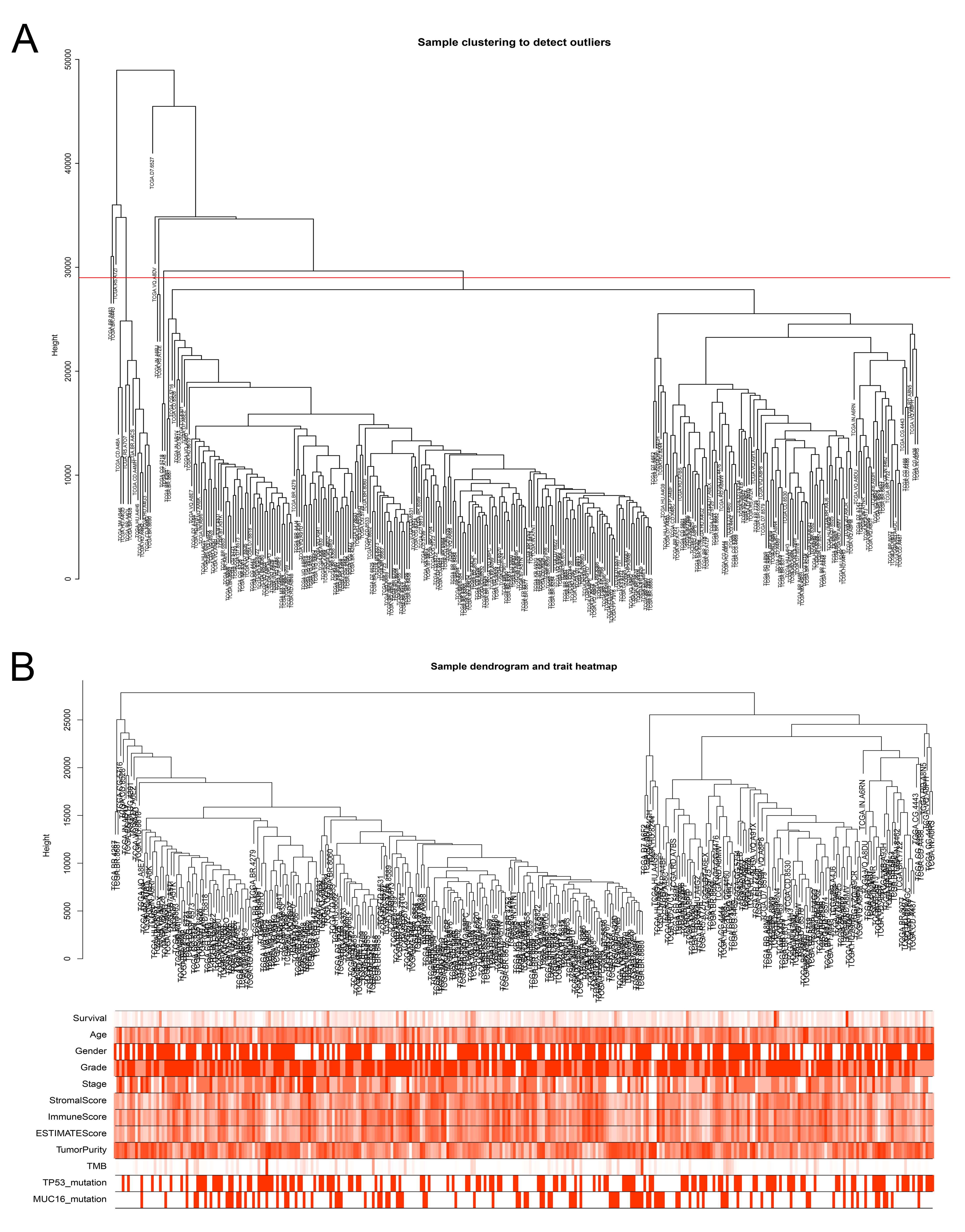

Supplement: FIGURE S2 — Sample clustering dendrogram. (A) Sample clustering was conducted to detect outlier samples. Outlier samples (n = 23) were eliminated. (B) Trait heatmap and sample dendrogram after outlier sample elimination. A total of 14 samples were included in the dendrogram. Color concentration is in proportion to survival, age, gender, stage, stromal score, immune score, ESTIMATE score, tumor purity, tumor mutation burden, TP53 mutation status, and MUC16 mutation status. [file Image_2.TIF]

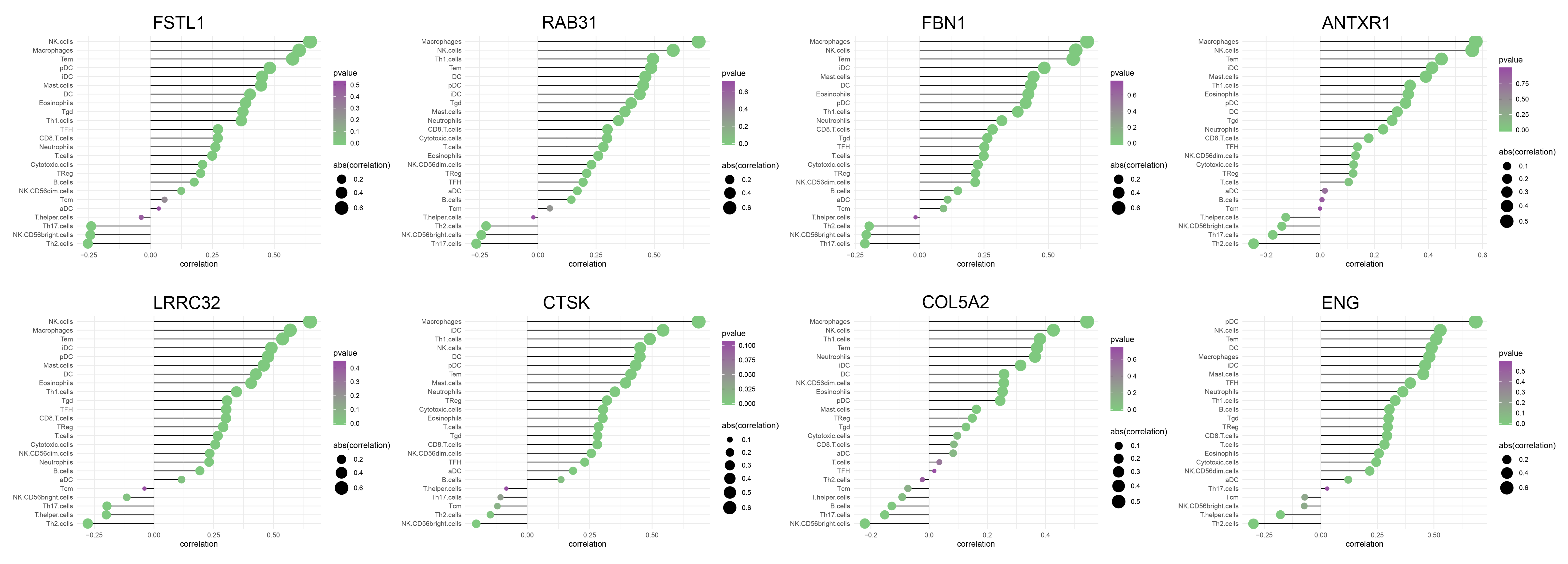

Supplement: FIGURE S3 — Correlation analysis of the eight hub genes with 24 immune signatures in ssGSEA. [file Image_3.TIF]

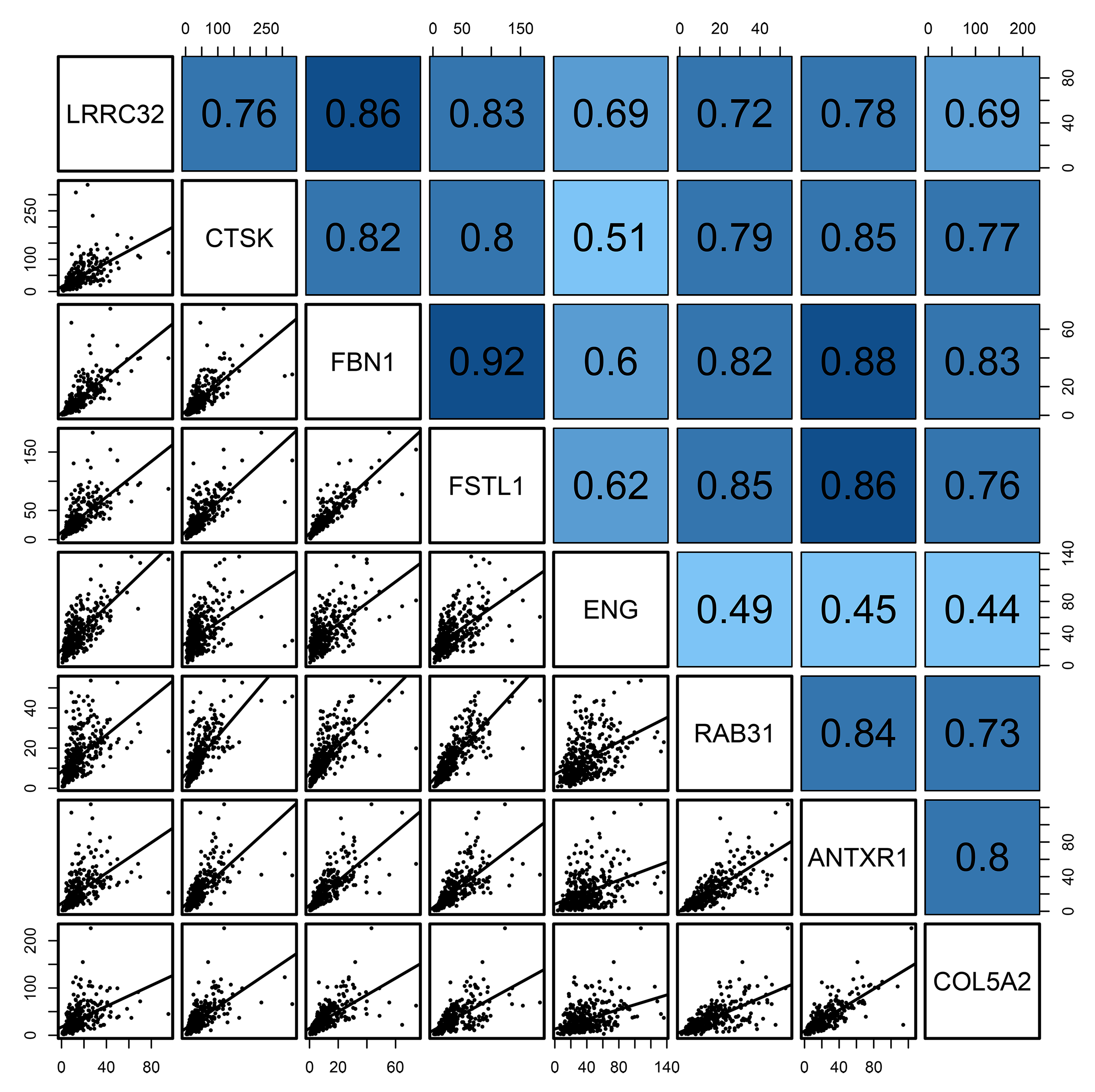

Supplement: FIGURE S4 — Co-expression analysis of the hub genes at transcriptional level. [file Image_4.TIF]
