# Supplementary material for: Gastric Cancer Tumor Microenvironment Characterization Reveals Stromal-Related Gene Signatures Associated With Macrophage Infiltration
Source: Front Genet. 2020 Jun 30;11:663. doi: 10.3389/fgene.2020.00663 (PMC7339942; doi:10.3389/fgene.2020.00663)
Supplement: TABLE S1 — Clinical features of STAD patients in TCGA database. [file Table_1.DOCX]

Supplementary Table 1. Clinical features of STAD patients in TCGA database

| Variable | Patients, n (%） |
| --- | --- |
| Sex |  |
| Male | 241 (64%) |
| Female | 134 (36%) |
| Age (years) |  |
| ≤65 | 164 (44%) |
| ＞65 | 211 (56%) |
| Grade |  |
| G1 | 10 (3%) |
| G2 | 137 (37%) |
| G3 | 219 (58%) |
| GX | 9 (2%) |
| Pathologic Stage |  |
| I | 53 (14%) |
| II | 111 (30%) |
| III | 150 (40%) |
| IV | 38 (10%) |
| unknow | 23 (6%) |
| Pathologic T |  |
| T1 | 19 (5%) |
| T2 | 80 (21%) |
| T3 | 168 (45%) |
| T4 | 100 (27%) |
| TX | 8 (2%) |
| Pathologic N |  |
| N0 | 111 (30%) |
| N1 | 97 (26%) |
| N2 | 75 (20%) |
| N3 | 74 (20%) |
| NX | 18 (4%) |
| Pathologic M |  |
| M0 | 330 (88%) |
| M1 | 25 (7%) |
| MX | 20 (5%) |
